# Transcription-associated DNA DSBs activate p53 during hiPSC-based neurogenesis

**DOI:** 10.1038/s41598-022-16516-5

**Published:** 2022-07-15

**Authors:** Nadine Michel, Heather M. Raimer Young, Naomi D. Atkin, Umar Arshad, Reem Al-Humadi, Sandeep Singh, Arkadi Manukyan, Lana Gore, Ian E. Burbulis, Yuh-Hwa Wang, Michael J. McConnell

**Affiliations:** 1 Neuroscience Graduate Program, University of Virginia School of Medicine, 1340 Jefferson Park Avenue, Charlottesville, VA 22908 USA; 2 Department of Biochemistry and Molecular Genetics, University of Virginia School of Medicine, 1340 Jefferson Park Avenue, Charlottesville, VA 22908 USA; 3 Sede de la Patagonia, Facultad de Medicina y Ciencias, Universidad San Sebastián, Puerto Montt, Chile; 4 Lieber Institute for Brain Development, 855 N. Wolfe St., Ste. 300, Baltimore, MD 21205 USA

**Keywords:** Developmental neurogenesis, Neural stem cells

## Abstract

Neurons are overproduced during cerebral cortical development. Neural progenitor cells (NPCs) divide rapidly and incur frequent DNA double-strand breaks (DSBs) throughout cortical neurogenesis. Although half of the neurons born during neurodevelopment die, many neurons with inaccurate DNA repair survive leading to brain somatic mosaicism. Recurrent DNA DSBs during neurodevelopment are associated with both gene expression level and gene length. We used imaging flow cytometry and a genome-wide DNA DSB capture approach to quantify and map DNA DSBs during human induced pluripotent stem cell (hiPSC)-based neurogenesis. Reduced p53 signaling was brought about by knockdown (p53^KD^); p53^KD^ led to elevated DNA DSB burden in neurons that was associated with gene expression level but not gene length in neural progenitor cells (NPCs). Furthermore, DNA DSBs incurred from transcriptional, but not replicative, stress lead to p53 activation in neurotypical NPCs. In p53^KD^ NPCs, DNA DSBs accumulate at transcription start sites of genes that are associated with neurological and psychiatric disorders. These findings add to a growing understanding of how neuronal genome dynamics are engaged by high transcriptional or replicative burden during neurodevelopment.

## Introduction

Evolution has devoted significant cellular resources to genome maintenance and the prevention of somatic mutations. Numerous DNA repair pathways exist to counteract the myriad of DNA lesions brought about by ongoing replication, transcription, and metabolism^1–4^. DNA damage signaling pathways promote DNA repair by engaging cell cycle checkpoints that stall cell cycle progression until repair is complete^5^. When DNA repair fails, persistent DNA damage signaling often initiates cell death^6^. Following from this general mechanistic model, DNA repair mechanisms have been thought of as cellular caretakers. DNA damage signaling mechanisms, in turn, play the role of cellular gatekeepers protecting an individual from potentially tumorigenic somatic mutations^7–9^. The tumor suppressor protein p53 is a prototypical gatekeeper, sometimes referred to as “the guardian of the genome”^10,11^.

Human development requires hundreds of billions of cell divisions to reach and maintain a steady-state level of ~ 10^15^ cells in an adult. Human neocortical development is additionally constrained by an absence of neurogenesis after birth^12,13^. The human cerebral cortex is comprised of 15–20 billion neurons that are among the longest-lived and most diverse mammalian cells^14^. These arise from a discrete neural progenitor cell (NPC) pool in the ventricular zone of the developing cerebral cortex^15^. NPCs overproduce neurons by ~ twofold, thus requiring at least 40 billion NPC divisions during a few months of development^16–20^. This presents an enormous burden for genome maintenance, notably in the NPC response to DNA double strand breaks (DSBs).

DNA DSBs are potent somatic mutagens. Mouse mutants that lack nonhomologous end-joining (NHEJ) DNA DSB repair proteins exhibit extensive NPC death that precludes neurodevelopment and perinatal survival^21–24^. However, NHEJ-deficient mice survive in a p53-deficient background, albeit with an increased occurrence of medulloblastoma and complex karyotypes^25,26^. A more recent study further demonstrates a clear role for p53 in the removal of aneuploid cells during mouse cortical development^27^.

Genetic neurodegenerative diseases are brought about by deficits in genome maintenance^28,29^. Motivated in part by many new findings of somatic mutations in neurons^30–35^, novel approaches to identify recurrent sites of DNA damage in neurons have been developed^36^. In addition to primary, typically post-mortem, tissue, human induced pluripotent stem cell (hiPSC)-based neurogenesis has proven to be a concordant and tractable model system. One DNA DSB mapping approach, high-throughput, genome-wide, translocation sequencing (HTGTS), uses a genome maintenance-deficient genetic background (e.g., XRCC4^−/−^P53^−/−^) to slow DNA DSB resolution dynamics and create endogenous “prey” sites that are captured at engineered “bait” sites via chromosomal translocation^37^. Further pharmacological and patient hiPSC-based interrogation using HTGTS finds that both DNA replication and gene transcription are endogenous sources of recurrent DNA DSBs^38–40^. Gene expression level and gene length also predispose subsets of neural genes to recurrent DNA DSBs^41–43^. Neuronal genes with this predisposition for somatic mutation are enriched for associations with neurodevelopmental and neuropsychiatric disease^44^; thus, these observations point to distinct, unresolved aspects of neuronal genome dynamics that potentially mediate neurogenetic architecture through brain somatic mosaicism.

Previously, we developed imaging flow cytometry (IFC)^45^ to quantify DNA DSBs with single cell resolution during hiPSC-based neurogenesis. This study revealed increasing levels of DNA DSBs during the first 2 weeks of hiPSC-based neurogenesis prompting us to further investigate neurogenic DNA damage signaling. Here, we sought to limit, rather than abrogate, p53 activity during this time and generated neurotypical NPCs that express short-hairpin RNA (shRNA) targeting p53 (p53^KD^) or a scrambled shRNA control. Marked increases in proliferation and survival accompanied increased DNA DSB burden during p53^KD^ neurogenesis. Genome-wide DNA DSB mapping^46^ identified an acute (24 h) DNA DSB increase in p53^KD^ NPCs that was associated with gene transcription, but not gene length. Interestingly, the only additive effect of replication stress (via aphidicolin) was observed in control NPCs after 4 days. This effect was associated with both gene transcription and gene length, and was mitigated by p53^KD^. Collectively, our work demonstrates a role for p53 signaling in the rapid resolution of transcription-associated DNA DSBs that are incurred during neurotypical human neurogenesis.

## Results

### p53^KD^ elevates DNA DSB burden in hiPSC-derived neurons

**Figure 1:**
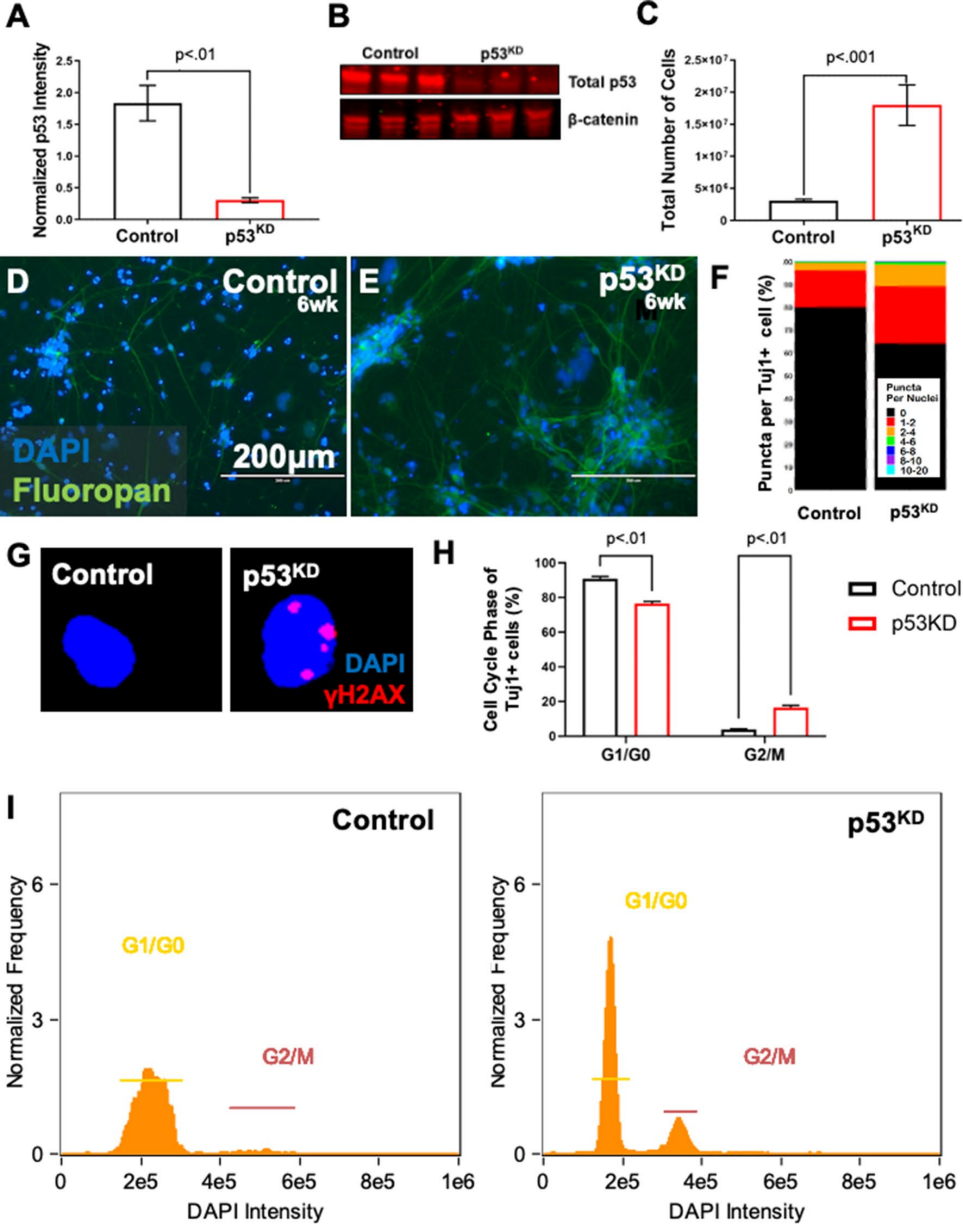
Neural p53^KD^ increases cell number, DNA DSBs, and mitotic activity. (**A**,**B**) Marked reduction in p53 protein in p53^KD^ neurogenesis (Unpaired t-test, *p* < .01). Blot is cropped, original blot presented in Supplementary Figure S7A. (**C**) After 6 weeks of neuronal differentiation, p53^KD^ cultures have > threefold more cells than control (Error Bars = Mean +/− SEM, unpaired t-test, *p* < .001). (**D**,**E**) Representative images of Control and p53^KD^ neuronal cultures stained with DAPI and Fluoropan (Neuron Specific Cocktail antibody). (**F**) Pooled stacked histogram of γH2AX puncta per nucleus from two neurotypic cell lines (BJ, 9429). p53^KD^ neurons (Tuj1 +) have significantly more DNA DSBs (N = 47,406, Chi-squared test, *p* < .0001). (**G**) Representative IFC images of neurons with γH2AX foci. (**H**,**I**) p53^KD^ (Tuj1 +) vs Control have fewer cells in G1/G0 (Two-way ANOVA, Tukey’s Test *p* < .01), and more cells in G2/M (Two-way ANOVA, Tukey’s Test *p* < .01).

Genomic loci that frequently incur DNA DSBs, such as fragile sites, are prone to chromosomal recombination^47,48^. Neuronal genes that harbor sites of recurrent DNA DSBs have been put forward as a mechanistic link to the consequences of brain somatic mosaicism^40^. We probed the role of p53 during hiPSC-based neurogenesis using a knockdown (KD) approach. NPCs from two neurotypic hiPSC lines (BJ and 9429, Fig. S1) were transduced to express a scramble short hairpin RNA (shRNA) or shRNA targeting p53 transcripts; p53^KD^ markedly diminished p53 protein relative to shRNA controls (Figs. 1A,B, S7A). When differentiated to cortical neuron fates, an abundance of cells was observed in p53^KD^ cultures relative to controls after 6 weeks (Fig. 1C–E).

DNA DSB levels were quantified at 6 weeks of hiPSC-based neurogenesis using IFC^45,49,50^. Immunoreactive γH2aX puncta were counted in single β-III tubulin-positive cells (i.e., neurons) using the TUJ1 antibody^51^. p53^KD^ cultures had more neurons with DNA DSBs (Fig. 1F,G). Given the increase in cell number observed in p53^KD^ cultures, we examined whether TUJ1-positive p53^KD^ cells were still mitotic. In control cultures, neurons were predominately in G1/G0 as expected; however, p53^KD^ cultures had a significant increase of TUJ1-positive cells in G2/M (Fig. 1H,I). This suggests that not all p53^KD^ TUJ1-positive cells are fully post-mitotic neurons.

### DNA DSBs accumulate upstream of p53^KD^ phenotypes

**Figure 2:**
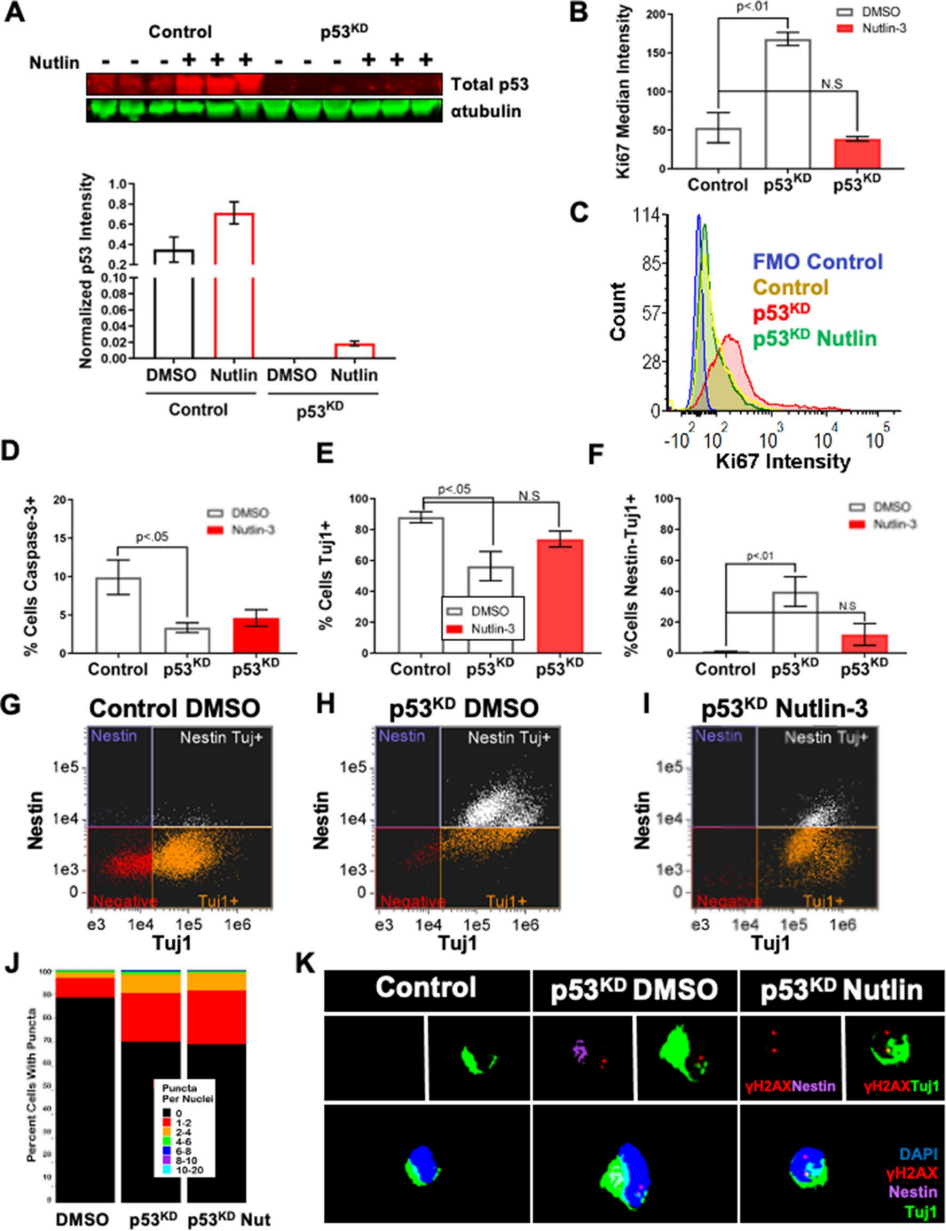
Nutlin treatment rescues p53^KD^ phenotypes in early neurogenesis. Nutlin treatment rescues delayed neurogenesis phenotypes in p53^KD^ at 1 week of neuronal differentiation. (**A**) One week treatment with 15 μM Nutlin-3 partially restores total p53 levels in p53^KD^ neural cells. Blot is cropped, original blot presented in Supplementary Figure S7B. (**B,C**) p53^KD^ neuronal cultures have more Ki67 + cells vs control and nutlin treated p53^KD^ (One-way ANOVA, Dunnett’s Test *p* < .01). (**D**) p53^KD^ have less cell death vs controls (One-way ANOVA, Dunnett’s Test, *p* < .05). (**E**) Control cells had the highest percentage of Tuj1 + cells vs p53^KD^ cells (One-way ANOVA, Dunnett’s test, *p* < .05). No significant difference between controls vs Nutlin p53^KD^ (One-way ANOVA, Dunnett’s Test *p* = .10)**. (F**) Nestin +/Tuj1 + cells were strikingly enriched in p53^KD^ cultures (One-way ANOVA, Dunnett’s Test *p* < .01). These account for the Tuj1 + cells in G2/M from Fig. 1. p53^KD^ cells treated with DMSO had significantly more double positive cells, nutlin treatment significantly reduced this population (One-way ANOVA, Dunnett’s Test, *p* = .51). (**G**,**H**,**I**) Representative imaging flow cytometry plots of samples of control, p53^KD^, and p53^KD^ treated with Nutlin. (**J**) Nutlin treatment did not reduce DNA DSB level in p53^KD^ vs control (*p* < .0001, KS test) (*p* < .0001, KS test). (**K**) Representative images from imaging flow cytometry.

Rescue studies were performed to identify p53^KD^ phenotypes that were dependent on p53 signaling. Neural differentiation experiments were supplemented with the small molecule Nutlin-3 which promotes p53 activation by inhibiting MDM2^52^. Notably, Nutlin treatment was lethal to control NPCs (Fig. S2), as observed in other cell types^53,54^. A modest increase in total p53 levels was observed in Nutlin-treated p53^KD^ NPCs (Figs. 2A, S7B). Concordant with an increased cell number observed in p53^KD^ after 6 weeks of differentiation (Fig. 1), after 1 week p53^KD^ showed higher median Ki67 staining (Fig. 2B,C) and reduced levels of active Caspase 3 (Fig. 2D) relative to control. Diminished levels of TUJ1-positive cells in p53^KD^ were replaced by a large population of cells that expressed both TUJ1 and the NPC marker Nestin (Fig. 2E–H). Nestin is neurofilament specific to NPCs^55,56^ and double positives indicate a transitioning population of NPCs that maintain proliferative potential^45,57^. Nutlin treatment restored Ki67 and TUJ1 phenotypes to control levels (Fig. 2B,C,E,I) and dramatically reduced the TUJ1/Nestin population (Fig. 2F,I). DNA DSB burden in p53^KD^ is more prevalent after 1 week relative to 6 weeks (Fig. 1F) of differentiation, and is unaffected by Nutlin treatment (Fig. 2J,K).

### Elevated DNA DSB burden in p53^KD^ NPCs is associated with gene expression level

Additional DNA DSBs in p53^KD^ NPCs likely arise due to endogenous stressors like transcription or replication. To address this question, we mapped unrepaired DNA DSBs with single nucleotide resolution using DNA DSB Capture with modifications^46,58^. Wang, et al. linked unrepaired DNA DSBs to NPC replicative stress and additional somatic mosaicism at autism risk genes using HTGTS to identify DNA DSB location after DNA DSBs were resolved via chromosomal translocation^39^. Their measurement was taken after 4 days of NPC expansion when treated with the DNA polymerase inhibitor aphidicolin (APH).

We reasoned that a p53 response may have faster kinetics, therefore we performed DSB mapping on control and p53^KD^ NPCs after both 1 and 4 days APH treatment. Biological replicates showed strong concordance and were pooled for subsequent analysis (Tables S1, S3A). Captured reads were mapped and DNA DSB location was assessed in 5 categories of genomic regions (promoter, transcription start sites (TSS), gene body, transcription termination sites (TTS), and intergenic) (Fig. S4A,B). DSB density was similarly distributed among all categories and cell conditions. Notably, across all conditions the TSS had the highest percentage of DSB density (Fig. S4A,B).

**Figure 3:**
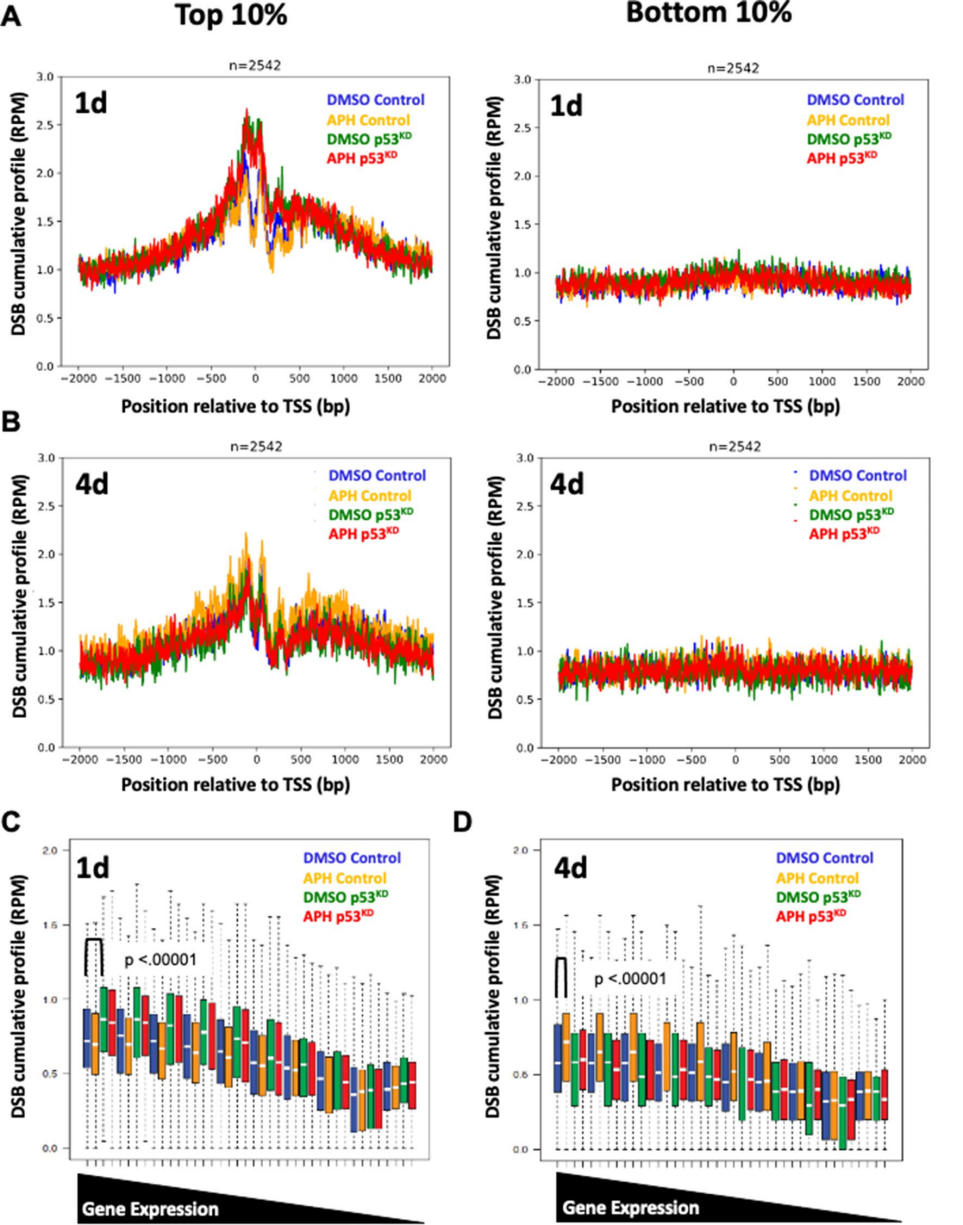
p53^KD^ leads to an acute increase in TSS-associated DSBs. In highly transcribed genes (n = 2542), DNA DSBs are enriched at the TSS of NPCs treated for 1 day (**A**) and 4 days (**B**). (**C**) Elevated DNA DSB levels in p53^KD^ NPCs are associated with gene expression levels in 1 day cultures. Boxplot of DSB coverage (TSS ± 500 bp) in 10 equal bins based on the gene expression. p53^KD^ NPCs have more DNA DSBs in highly transcribed genes compared to DMSO Control cells (Wilcoxon signed-rank test, two-sided, paired test, *p* < .00001). (**D**) After 4 days APH treatment, control NPCs treated with APH now have more DNA DSBs compared to untreated, p53^KD^, and p53^KD^ treated with APH in highly transcribed genes (Wilcoxon signed-rank test, two-sided, paired test, *p* < .00001). The box plot shows the 25th and 75th percentile; the middle line is the median; the whiskers span 5% to 95%, and outliers are not shown.

In other cell types the TSS of highly expressed genes incurs DNA DSBs^46,59–61^, therefore, we examined this in NPCs. Relative gene expression levels were binned into 10 subsets (n = 25,420 total genes) from control scRNA seq data reprocessed as bulk (Fig. S5E). When we fine-mapped to TSS +/− 2 kb, accumulation of DSBs was observed in the top 10% of expressed genes compared to the bottom 10% of expressed genes in both WT and p53^KD^ across treatment conditions (Fig. 3A,B). Consistent with elevated DNA DSB burden in p53^KD^ neurons (Figs. 1F,G and 2J,K), we captured significantly more DNA DSBs at TSSs in p53^KD^ NPCs compared to control, regardless of APH treatment, after 24 h of NPC expansion (Fig. 3C). However, no difference was observed as a result of p53^KD^ after 4 days (Fig. 3D).

Many neuronally-expressed genes are among the longest genes in the human genome^43,44^. Long genomic loci are transcribed into S-phase leading to potential collisions between RNA and DNA polymerases and ensuing DNA DSBs^42^. However, we found no effect of p53^KD^ on DNA DSB location with respect to gene length at either time point (Fig. S3B, C). An association between DNA DSBs and gene length was observed in control cultures treated with APH for 4 days (Fig. S3B), as described in previous studies^39^. Importantly, our DSB mapping approach is both genome-wide and unbiased; it is not restricted to translocations with defined bait sites as in HTGTS. This finding strengthens accumulating evidence that NPC replication stress leads to recurrent DNA DSB sites at long genes. However, p53^KD^ precludes the effect of 4 day APH treatment at both long and highly expressed genes (Figs. S3C and 3D).

Given no additive effect of APH treatment on DNA DSB levels in p53^KD^ NPCs and no association between gene length and recurrent p53^KD^ DNA DSBs, these data indicate that p53 may mediate an acute (24 h) response to DNA DSBs associated with high transcriptional activity. And that transcription-associated DNA DSBs in p53^KD^ NPCs are resolved at the population level during 3 additional days of NPC expansion. Taken together, genome-wide DNA DSB mapping data suggest that elevated DNA DSB burden in p53^KD^ NPCs is due to a diminished p53 response to transcriptional, but not replicative, stressors.

### p53 is activated during TOP1-mediated stress in hiPSC-derived NPCs

In hyperproliferative autism spectrum disorder patient-derived NPCs, replicative stress activates ATR with minimal p53 activation^39^. Given that p53^KD^ leads to an acute increase of recurrent DNA DSBs at TSSs, we sought to define p53 activation in response to replicative or transcriptional stress. We treated control NPCs with APH or with a topoisomerase I inhibitor camptothecin (CPT)^62^.

**Figure 4:**
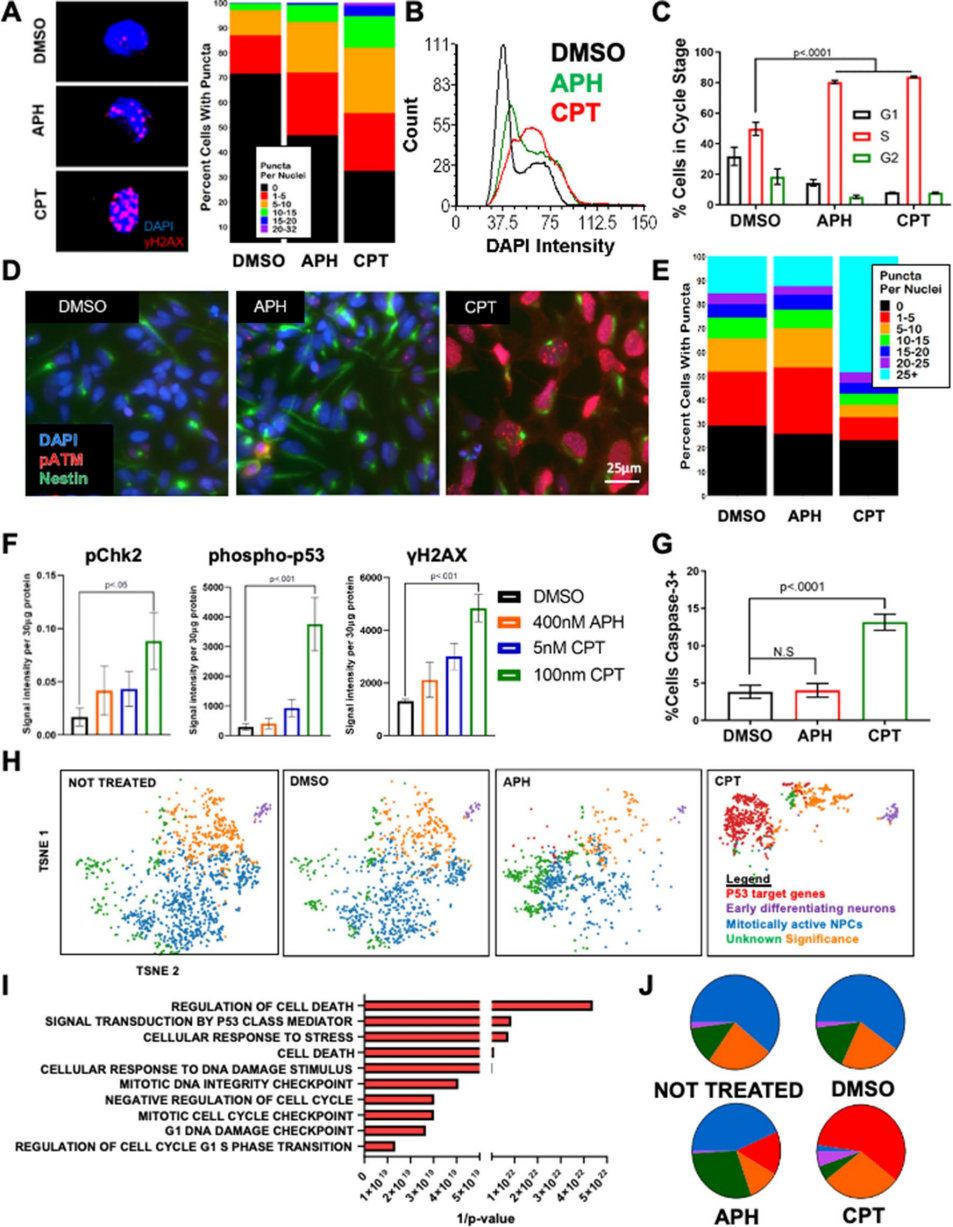
TOP1 inhibition elicits a robust p53 response in human NPCs. (**A**) NPCs treated with either 400 nM APH or 100 nM Camptothecin (CPT) had significantly more DNA DSBs per nucleus (Chi-squared, *p* < .0001, N = 63,436 cells). CPT cells had more DSB/cell (KS test, *p* < .0001, N = 63,436 cells). (**B**,**C**) NPCs exposed to either APH or CPT show significant S-phase arrest (Two-way ANOVA, Dunnett’s Test *p* < .0001). (**D**,**E**) NPCs have more pATM foci per nucleus after CPT treatment (One-way ANOVA, Dunnett’s Test, *p* < .0001) and less pATM foci per nucleus in replication stress (APH) (One-way ANOVA, Dunnett’s Test, *p* < .01). (**F**) Western blots identify elevated pChk2 (unpaired t-test, *p* < .05), phosphorylated p53 (unpaired t-test, *p* < .001), and γH2AX (unpaired t-test, *p* < .001). (**G**) Caspase-3 positive cells were elevated only in CPT treated NPCS (One-way ANOVA, Dunnett’s Test *p* < .0001) (One-way ANOVA, Dunnett’s Test, *p* = .98). (**H**) Single-cell RNA seq reveals that CPT-treated NPCs have differential expression of p53 target genes involved in DNA damage response, cell cycle arrest, and cell death (N = 4598, n is number of cells). TSNE plots shown for each condition. TSNE 1 and TSNE 2 are the same for each condition. (**I**) Functional pathway analysis of the top 100 highest variant genes from the 592 cells in the red cluster (enriched in CPT treatment) are involved cellular processes related to DNA damage checkpoint (*p* < 1.37E−22), cell death (*p* < 1.16E−22), and p53 mediators (*p* < 1.16E−22). (**J**) The percentage of cells in each cluster per condition is shown in a pie chart.

Topoisomerase I (TOP1) relieves DNA supercoiling due to ongoing transcription and replication by transiently binding to the DNA, forming a cleavage complex (TOP1cc), and then catalyzing single strand cleavage. Inhibition of TOP1 using camptothecin has revealed that TOP1 plays an important role in the elongation stage of transcription at long neural genes that could contribute to somatic mosaicism in neurons^63^. Neuronal deletion of *Top1* in mice leads to an increase in DNA DSBs, elevated somatic mosaicism, and early onset neurodegeneration^64^. Ataxia telangiectasia (A-T) patient-derived neurons are sensitive to CPT treatment^63^. We used identified concentrations of APH and CPT that increased the percentage of γH2AX-positive NPCs (Fig. S5A). Relative to controls, both APH and CPT treated NPCs had more total DNA DSBs (Figs. 4A, S5B) and significant S-phase arrest (Figs. 4B,C, S5C).

A canonical p53-dependent DNA DSB response includes phosphorylation of ATM, Chk2, and p53^65–67^. CPT-treated NPCs showed robust phosphorylation of ATM compared to controls and APH-treated NPCs (Fig. 4D,E). In addition, transcriptionally stressed NPCs had higher levels of phosphorylated p53, pChk2, and γH2AX compared to controls and replication stressed NPCs (Figs. 4F, S5D, S7C). NPCs exposed to replication stress had similar levels of cell death (assessed by active Caspase 3) relative to controls, but this population was more prevalent in TOP1 stressed cells (Fig. 4G). This was also observed when CPT and APH treatment led to more similar distributions of γH2AX puncta per nucleus (Fig. 4A, 9429, Figure S5B).

To further define the NPC response to TOP1 inhibition we performed single-cell RNA sequencing (scRNA-seq). We observed distinct phenotypic clusters present in all conditions (Fig. 4H, S5E). Actively proliferating NPCs (blue cluster) were abundant in the two controls (Not Treated, DMSO) and APH treated cells, but minimally present in the CPT treated NPCs (Figs. 4H,J, S5E). CPT treated cells had a significant emergence of NPCs upregulating genes involved in the DNA damage checkpoint, p53 mediators, and cell death (Fig. 4I). Consistent with increased cell numbers observed during p53^KD^ neurogenesis (Fig. 1C), these data confirm that TOP1 inhibition, but not replicative genomic stress, leads to a robust p53-mediated NPC response to DNA DSBs associated with increased cell death.

### Enrichment of DNA DSBs in p53^KD^ NPCs at the TSS of genes with clinical risk for neuropsychiatric disorders

**Figure 5:**
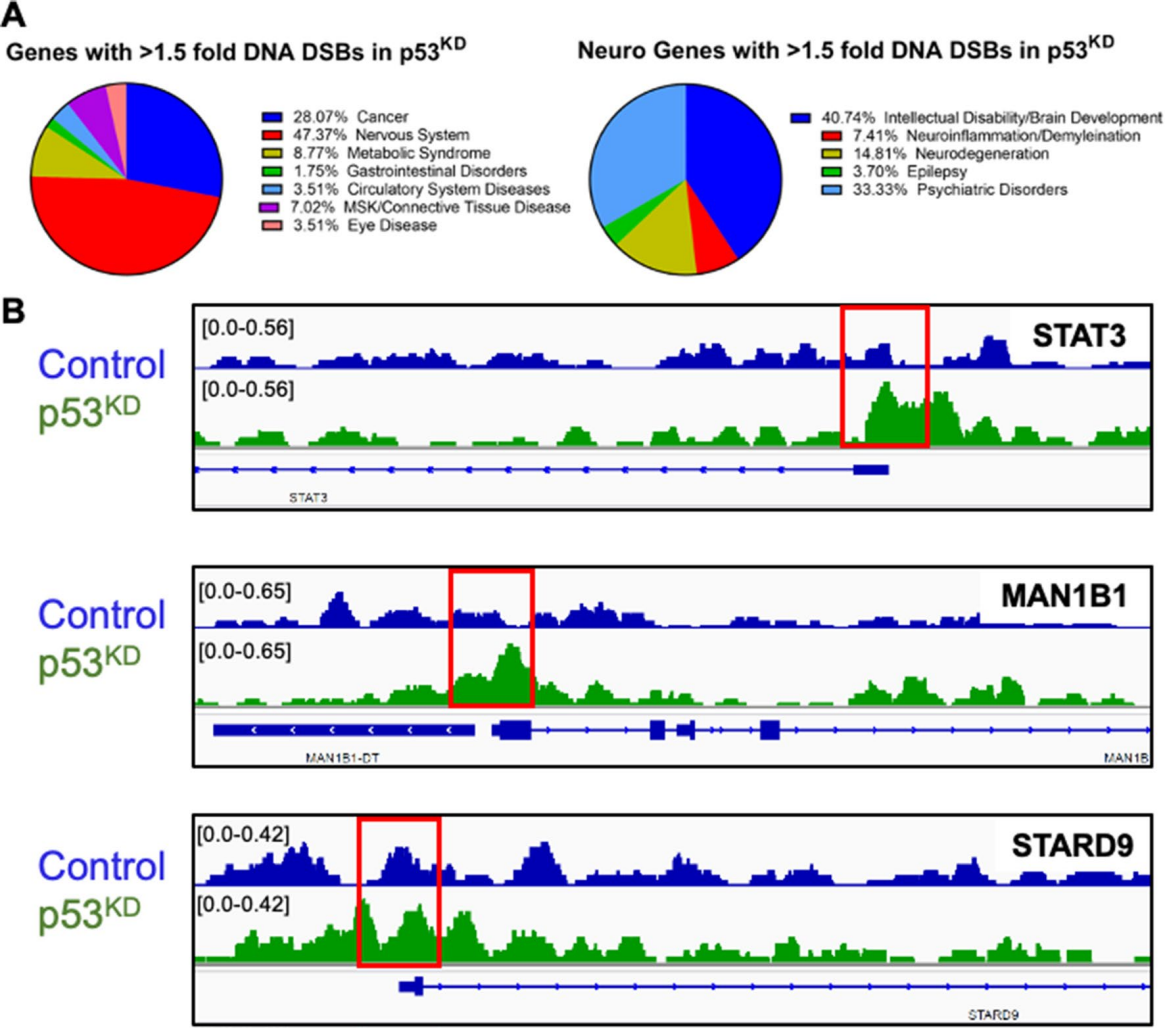
Recurrent sites of p53-susceptible DNA DSBs are associated with neurodevelopment and psychiatric disease. (**A**) 61 genes had > 1.5 fold DNA DSB increase in p53^KD^ NPCs vs control and were analyzed for known associations with disease. 47% of these genes are involved in the nervous system^80^. Among them, more than 40% play role in intellectual disability and 33% in psychiatric disorders (right). (**B**) An IGV plot of three genes associated with disorders of the nervous system showing an increase in DNA DSBs in p53^KD^ vs controls are shown (from top to bottom: STAT3, MAN1B1, STARD9). Red boxes highlight the TSS where the differences in DNA DSBs were quantified.

We sought to examine whether p53^KD^ increased the prevalence of DNA DSBs in genes with neuronal function or causal links to brain disease. We performed gene ontology (GO) analysis of expressed genes > 1.5 fold enrichment at TSSs in p53^KD^ NPCs. Of the 61 genes with enriched breaks, 47% were associated with disorders involving the nervous system (Fig. 5A, left). We analyzed these genes further and observed that 33% were involved in psychiatric disorders, ~ 15% in neurodegeneration, and more than 40% in intellectual disability and brain development (Fig. 5A, right). Specifically, recurrent DNA DSBs in p53^KD^ NPCs were enriched at STAT3 (Multiple Sclerosis^68^), MAN1B1^69^, and STARD9 (Bipolar Disorder^70^) (Fig. 5B, Table 1). We sought to determine whether the enrichment observed in the TSS of p53^KD^ NPCs was simply a function of these genes being selectively transcribed during neurogenesis, however, we observed that expression of the identified neuronal genes was not driving DSB enrichment (Fig. S6A–C). Together these data show that a lack of p53 is associated with recurrent DNA DSBs in the TSS of genes that are important for neurotypical brain development.

**Table 1:** Top 10 genes with > 1.5-fold DNA DSB enrichment in p53^KD^ treated for 24 h.

Rank	Gene name	DNA DSB fold change	Function	Disease association	Overlapping gene(s)
2	STAT3	3.2	Controls cell proliferation, migration, and apoptosis	Multiple Sclerosis	
3	MAN1B1	3.1	ER protein that disposes misfolded glycoproteins	Rafiq Syndrome (Intellectual Disability, impaired motor movement, facial dysmorphism)	
5	KAT6B	2.9	Histone acetyltransferase	Ohdo Syndrome (Intellectual Disability, ptosis, narrow eye openings)	SNORD172
7	STARD9	2.7	Regulates the assembly and stability of both interphase and spindle microtubules	Bipolar Disorder	
8	VPS35	2.7	Creates mitochondria-derived vesicles, directs degradation of mitochondrial proteins	Parkinson’s Disease	
9	FIZ1	2.6	Zinc finger protein that regulates hematopoietic and lymphoid cells	Bardet-Biedl Syndrome (Intellectual Disability, polydactyly, renal anomalies, obesity, retinopathy)	
12	B9D1	2.5	Located in transition zone of cilia and prevents diffusion of proteins	Joubert Syndrome (Cerebellar and Brainstem Malformation)	
14	SGK3	2.3	Regulates ion channels, cell growth, proliferation, membrane transporters, survival, migration	Alzheimer’s Disease	
15	SIX5	2.3	Transcription factor	Alzheimer’s Disease	
18	CIPC	2.2	Regulates circadian rhythms	Cohen Syndrome (Intellectual Disability, microcephaly, hypotonia, developmental delay)	

The top 10 genes with > 1.5-fold DNA DSB enrichment related to brain disorders are listed from highest to lowest DNA DSB fold change.

## Discussion

A deeper understanding of human neuronal genome dynamics is motivated by increasing recognition that both neurodevelopmental and neurodegenerative human diseases are linked to genome maintenance. The abnormal accumulation of DNA damage is a common pathological feature among neurodegenerative diseases including Alzheimer’s disease^71,72^, Parkinson’s disease^73,74^, and Amyotrophic Lateral Sclerosis (ALS)^75,76^ and is correlated with normal aging^28,77^. DNA repair is not perfect and somatic mutations accumulate in neurotypical human neurons^78^, higher frequencies of somatic mutations are observed in neurodegenerative disorders^79^. Indeed, genome maintenance is critical in neurons and enhanced DNA repair is a pharmacological target to ameliorate cognitive decline^28,29^. Moreover, mutations affecting DNA repair and damage signaling (e.g., Atm) lead to childhood onset neurodegenerative disease (e.g., ataxia-telangiectasia)^80^ and have emerged as critical mediators of Huntington’s Disease onset^81^.

The tumor suppressor p53 is a central mediator of genome maintenance. Our findings are consistent with observations from different cell lines in which p53 participates in DNA damage response by activation of ATM-pChk2 pathway, cell cycle arrest, and cell death^82–84^). We have shown that p53 is similarly activated when DNA damage occurs in hiPSC-derived NPCs. Reduced p53 levels during hiPSC-based neurogenesis led to elevated levels of DNA DSBs in differentiating neurons. Nutlin treatment rescued p53-dependent phenotypes, but elevated DNA DSB levels persisted. Our mapping of recurrent DNA DSBs resulted in three primary findings supporting the overall conclusion that transcription-associated DNA DSBs activate p53 during hiPSC-based neurogenesis. First, unresolved DNA DSBs map to the TSS of transcribed genes, and these are more abundant in p53^KD^. Second, replication stress had no additive effect on recurrent p53^KD^ DNA DSB sites. Third, DNA DSBs brought about by transcriptional stress, but not replication stress, activate p53 during neurotypical hiPSC-based neurogenesis. Additional support for the veracity of our observations is reported when NPCs are stressed for 4 days with APH. As observed previously using other approaches^39^, we also report an association between gene length and recurrent DNA DSBs after long term replication stress.

Other recent studies suggest divergent roles for p53 in stem cell fate decisions during human pluripotent stem cell-based neurogenesis. Impaired neurogenesis is observed from p53 knockout (p53^−/−^) human embryonic stem cells (hESCs) and from p53KD hiPSCs^85,86^. Disorganized neuroepithelial rosettes were observed in neurodifferentiated p53^−/−^ hESCs^86^, and likewise limited neurogenesis was observed in p53^KD^ hiPSC-derived brain organoids^85^. By contrast, exacerbated neurogenesis was observed when Marin Novarro, et al*.* initiated p53^KD^ after p53-competent hiPSCs were differentiated to neuroepithelial stem cells. SImilar to our study, cortical organoid differentiation with p53^KD^ in this stem cell population was found to promote neurogenesis. Despite different approaches (i.e., p53^KD^ in established NPCs (passage 5 and 6) and 6 week 2D cortical neuron differentiation), we also find that p53^KD^ leads to an abundance of TUJ1 positive neurons, a 2 -threefold increase in total cell numbers, and diminished cell death. However, we also observe that many TUJ1-positive cells remain Nestin-positive, and are not post-mitotic. This neural population has been observed during hiPSC-based neurogenesis before^45,87^, and here we show that Nutlin treatment during the first week of p53^KD^ neurogenesis limits prolonged Nestin expression and mitosis in a subset (~ 20%) of TUJ1-positive cells. Although not conclusively established here, these data are consistent with a model wherein p53 activity promotes cell cycle exit and terminal differentiation of NPCs into post-mitotic neurons.

Collectively, our data indicate a distinct neurodevelopmental role for p53 in the response to transcription-associated DNA DSBs. It has been proposed, based on several lines of evidence, that collisions between DNA and RNA polymerases lead to DNA DSBs and subsequent somatic mosaicism at a subset of neuronal genes that are long and are also risk alleles for psychiatric disease^37,40,42^. Consistent with this mechanism, we find an association between gene length and DNA DSB prevalence in neurotypical NPCs. However, it is only when considered with regard to gene expression level that we observe a differential incidence of DNA DSBs in p53^KD^ NPCs. Furthermore, our DNA DSB fine-mapping approach found increased levels of TSS-associated DNA DSBs in p53^KD^ NPCs. The TSS has previously been shown to have enriched DSB density compared to other genomic regions^46,59–61^. Elevated DNA DSB levels in p53^KD^ could be an indirect consequence of diminished p53-dependent transcriptional activation^88^, or these could arise as a direct consequence of impaired p53 recruitment to sites of DNA damage^89^. Further investigation is required to determine if elevated TSS-associated DNA DSBs are a direct or indirect consequence of p53^KD^.

Longitudinal molecular genetic and biochemical studies of human neurodevelopment are intractable. Cross-sectional familial and population-based genetic studies collectively conclude that most human neurological disease is polygenic and challenging to interpret. Studies of mouse mutants together with studies of post-mortem human brains have yielded many insights; yet the translation of mouse data to human disease is imperfect. This barrier to progress is brought about both by species-specific brain differences and by the end-stage nature of most human post-mortem tissue. The study of human neurodevelopment in-a-dish is enabled by hiPSC-based neurogenesis. In addition to providing mechanistic insight and drug development platforms for human neurological disease^90–95^ hiPSC-based models offer insights into neurotypical neurodevelopment that are uniquely human. Given dramatically different neuronal production requirements and human-specific brain phenotypes, it is not surprising that brain somatic mosaicism is more abundant in human brains^30,32^, relative to mouse^35,96^. A human-specific understanding of the interplay between endogenous genomic stress and genome maintenance responses portends new drug targets for neuropsychiatric and neurodegenerative disease.

## Experimental procedures

### Cell culture

Fibroblasts from neurotypical individuals were reprogrammed to induced pluripotent stem cells using lentiviral (9429) or non-integrating Sendai (Invitrogen) vectors^12^. NPCs and neurons were generated following methods outlined by Brennand and Livesey^91,97^. Briefly, hiPSC-derived NPCs were plated on Matrigel (Fisher) or Poly-Ornithine (Sigma)/ Laminin (Invitrogen) and passaged 1:6 every 4 days. NPCs (< passage 14) were cultured in NPC medium (DMEM/F12 + Glutamax (Invitrogen), 1 × N2 (Invitrogen), 1X B27-Vitamin A (Invitrogen), 1ug/ml Laminin (Invitrogen), 20 ng/ml FGF-2 (Peprotech) and dissociated in Accutase (Fisher) for 5 min at 37 °C. hiPSC-derived neurons were cultured on Matrigel in Neuron Medium (DMEM/F12 + Glutamax, 1 × N2, 1X B27 with Vitamin A (Invitrogen), 20 ng/ml BDNF (Shenandoah Biotechnology), 20 ng/ml GDNF (Shenandoah Biotechnology), 1 mM dibutryl-cyclic AMP (Sigma), and 200 nM ascorbic acid (Stem Cell Technologies).

### Drug treatment

NPCs were exposed to various doses of Aphidicolin (Sigma), Camptothecin (Sigma), and Nutlin-3 (Sigma). DMSO was used to solubilize all drugs and was used as a vehicle control. For 1 day APH treatments, cells were passaged and the next day treated with 0.4 μM. In 4 days APH treatments, cells were passaged and treated 2 days later. Treatment consisted of 3 days of 0.5 μM APH and 1 day of 0.25 μM APH as outlined by Wang et al.^39^. All treatments were added directly to the appropriate cell media. All drugs used in this study were stored appropriately at − 20 °C and aliquoted.

### Immunocytochemistry

NPCs were cultured for 24 h with various drugs and rinsed with PBS. NPCs at ~ 80% confluency were fixed on 18 mm glass coverslips with 4% PFA for 15 min at room temperature and rinsed (3 times for 5-min washes) in PBS. Cells were permeabilized with 0.5% Triton-X 100 for 10 min and incubated in blocking buffer (2% BSA in PBS-T (0.1% Triton-X 100)) for one hour at room temperature. The cells were probed overnight at 4 °C with anti-phosphorylated ATM (pATM), DAPI, and Nestin followed by labeling with secondary staining and incubated for one hour at room temperature. pATM foci were quantified as described in our previous studies^45^. In short, NPCs were imaged on a fluorescent EVOS Microscope and analyzed through a Cell Profiler (Broad Institute) pipeline that (1) isolates channels from an RGB image, (2) identifies the nuclei^98^, identifies foci within the nucleus, and (4) quantifies the number of foci per nucleus.

### Western blots

NPCs were dissociated with Accutase, pelleted, flash frozen with liquid nitrogen, and stored at − 80 °C. Cells were lysed with 2 × Laemmli buffer (4% SDS, 10% 2-mercaptoethanol, 20% glycerol, 0.004% bromophenol blue, 0.125 M Tris HCl, pH 6.8) and boiled for 10 min at 95 °C. For immunoblotting, total cell lysate was resolved by 4–15% Mini-PROTEAN precast gradient gel (Biorad). Proteins were transferred to a 0.2 μm nitrocellulose membrane for 90 min at 100 V and incubated at room temperature for 1 h in blocking buffer (1% Fish Gelatin (Sigma) in TBS-T (Tris-buffered Saline 0.1% Tween-20)). The blots were incubated at 4 °C overnight in blocking buffer in primary antibody and incubated for 45 min at room temperature in secondary antibody. Blots were visualized on a Licor Odyssey Imaging System. All gels were run with a positive control (High dose CPT treated HEK 293 T cells). Differences in abundance of protein in loading controls (αtubulin, actin, β-catenin) between samples exposed to genomic stress were observed; therefore, samples were normalized by loading equivalent protein (30 μg) in each lane (as determined through BCA assay). The following well characterized primary antibodies were used for studies included in this publication: Total p53^99^, phosphorylated-p53^98^, pChk2^100^, αtubulin^101^, β-catenin^102^, Total H2Ax^103^, and γ-H2AX^104^.

### Single-cell RNA sequencing

NPCs were exposed to either 400 nM APH, 300 nM CPT, DMSO (control), or no treatment for 24 h. High viability cell samples collected for scRNA-seq using the 10 × Genomics V2 Kit for reverse transcription and library preparation. Sequencing was performed on the Next-seq 500 and FASTQ files were run through the cellranger pipeline and further analyzed through loupe browser software and software packages in R program. Gene ontology analysis was performed using Panther. scRNA-seq data can be accessed at the NCBI Sequence Read Archive (SRA), accession number: PRJNA591220.

### Wildtype bulk gene expression analysis

To evaluate overall gene expression relationship with DSBs, scRNA-seq data from the wildtype cell line was aggregated and processed using the Tuxedo suite^105^. Genes with FPKM > 0 were evenly divided into 10 bins for further analysis with genome-wide DSB mapping and sequencing data.

### P53 knockdown NPCs

hiPSC-derived NPCs were transfected with a lentivirus containing a puromycin resistant plasmid containing a vector backbone control (pLKO.1) or p53 shRNA containing plasmid (AdGene). After 24 h of exposure to the lentivirus, NPCs were changed to normal NPC media for 24 h and puromycin selection for 4 days at 0.4 μg/mL. Knockdown efficiency was confirmed with Western Blot.

### Cell counts

Cell counts were performed for every sample to quantify the number of cells remaining in culture and accurately add the appropriate dilution of antibody for flow cytometry experiments. NPCs and Neurons were dissociated with accutase, spun down, and filtered through a 40 μm strainer into fixative solution (2% PFA in PBS) to obtain single cell suspensions. Cells were then counted using an automated Cell Countess system (Thermo Scientific). Live cell counts were also obtained using the Cell Countess system with Trypan Blue to distinguish between living and dead cells.

### Flow cytometry and imaging flow cytometry

NPCs and Neurons were collected, fixed, and stained for flow and imaging cytometry as outlined in detail in our previous study^45^. Samples were stored at 4 °C in the dark until they were run through the Imagestream Mark II System (Amnis Inc/Luminex Corp.) or a Cytek modified FACSCalibur™ cytometer. Single stain controls were used for compensation and gating was determined through fluorescence minus one (FMOs) controls and isotype controls. For flow cytometry experiments gating and analysis were performed using FCS Express 6 (DeNovo Software). Imaging flow cytometry data were analyzed using IDEAS® software (version 6.2). γH2AX foci were quantified using an efficient foci pipeline and masking strategy^45,49^. Cell cycle statistics were acquired using *Modfit* software. See supplemental information for more details on cytometry methods.

### Genome-wide break mapping and sequencing

Detection of DNA breaks was performed as previously described^58^. Briefly, fixed nuclei of the p53^KD^ and the control samples of NPC 9429 with and without APH treatment were subjected to blunting/A-tailing reactions, and Illumina P5 adaptor ligation to capture broken DNA ends. Genomic DNA was then purified and fragmented by sonication, and subsequently ligated to Illumina P7 adaptor, and the libraries were PCR-amplified for 15 cycles. Prepared libraries were then subjected to whole-genome, 75-bp and 150-bp paired-end sequencing with the Illumina NextSeq 500 and the HiSeq X Ten platforms, respectively. Two biological duplicates were performed for each sample treated for 1 day.

### DSB read processing

Sequencing reads were aligned to the human genome (GRCh38/hg38) with bowtie2^106^(v.2.3.4.1) aligner running in high sensitivity mode. Restriction on the fragment length from 100 to 2000 nt (-X 2000 -I 100 options) was imposed. Unmapped, non-primary, supplementary and low-quality reads were filtered out with SAMtools^107^(v. 1.7) (-F 2820). Furthermore, PCR duplicates were marked with picard-tools (v. 1.95) MarkDuplicates, and finally, the first mate of non-duplicated pairs (-f 67 -F 1024) were filtered with SAMtools for continued analysis. For each detected break, the most 5’ nucleotide of the first mate defined the DNA break position. Sequencing and alignment statistics for the DSB mapping/sequencing liubraries are listed in Table S1. Downstream data analysis following DSB read processing has been performed with BEDtools^108^(v. 2.27.1) and standard Linux commands to compute coverages. Results were visualized in Python3 (v. 3.6.5) with matplotlib (v. 2.2.2), numpy^109^ (v. 1.15.0), pandas^110^(v. 0.23.3), and R statistical software (v.3.4.4).

### DNA DSB gene analysis

Sixty-one genes had > 1.5-fold enrichment at TSSs +/− 250 bp in p53^KD^ NPCs treated for 1 day. These genes were examined using Panther for gene function and GeneCard for clinical risk/association. The genes were further categorized based on clinical risk and disease type. These 61 genes were then coded as either neuronal or non-neuronal related and the gene expression in each class was compared to the bulk gene expression distribution from wildtype cells.

### Genomic regions annotation

To assign genomic annotations BEDtools (v. 2.27.1) intersect was used to sequentially assign genomic features with each single-nucleotide break only being assigned to one genomic feature. The sequential feature assignment filters out breaks as they are assigned to a feature. The order for assigning genomic features was TSS, promoter, TTS, gene body, and those not assigned to any of these features are coded as intergenic. The GRCh38/hg38 build RefSeq genes were downloaded from the UCSC browser. The definitions used for each genomic feature is as follows: promoter region ranging from TSS − 1000 nt to − 250 nt, TSS region ranging from TSS − 250 to + 250 nt, gene body region ranging from TSS + 250 nt to TTS − 250 nt, and TTS regions ranging from TTS − 250 nt to + 250 nt. Counts in each genomic feature were then normalized to the megabase size of the genomic feature (breaks/Mb). The normalized density for breaks in each genomic feature were then plotted using Python (v. 3.8.10) and matplotlib.pyplot (v. 3.1.2).

### Statistical analyses

For all experiments, data are shown as mean +/− standard error of the mean of three to six biological replicates per cell line. At least 2–3 neurotypic cells lines were used per experiment and pooled together for further analysis. Statistical significance was determined by using an unpaired t-test, one-way ANOVA and Dunnett post hoc test for differences, or two-way ANOVA when applicable with a Sidak Multiple Comparison Test. DNA DSBs were plotted as stacked histograms and analyzed using a contingency table and the Chi-squared test for two samples and nonparametric KS test for greater than three samples.

### Accession numbers

DSB mapping and scRNA-seq datasets generated in this study can be accessed at the NCBI Sequence Read Archive (SRA) under the accession number: PRJNA591220. Flow cytometry data will be made available through Community Cytobank upon publication.

## Supplementary Information

Supplementary Information.
