# Supplementary material for: Transcription-associated DNA DSBs activate p53 during hiPSC-based neurogenesis
Source: Sci Rep. 2022 Jul 15;12:12156. doi: 10.1038/s41598-022-16516-5 (PMC9287420; doi:10.1038/s41598-022-16516-5)
Supplement: Supplementary file 1 — Supplementary Information. [file 41598_2022_16516_MOESM1_ESM.pdf]

Michel, et al. "Transcription-associated DNA DSBs activate p53 during hiPSC-based neurogenesis"

Supplementary Materials

Supplemental Experimental Procedures

Flow Cytometry

Flow cytometry was conducted on a Cytek modified FACSCalibur™ using the 488 Blue, 637 Red, and 407 Violet lasers to excite Ki-67 (PE), emission collected with a 580/20 filter, γH2AX (AlexaFluor 647) with a 661/16 filter, and DAPI with a 450/50 filter respectively. 15,000 cells were collected per sample at low pressure. Single stain controls were used for compensation and gating was determined through fluorescence minus one (FMOs) controls, isotype controls, and unstained controls. FCS Express 6 (DeNovo Software) was used to perform gating and analysis. Cell cycle was analyzed using statistical modelling from the program *Modfit*.

Imaging Flow Cytometry

Imaging flow cytometry (IFC) was performed using the Imagestream Mark II System (Amnis Inc/Luminex Corp.). Cells were collected, using INSPIRE® software, for 10 minutes or until 10,000 cells were acquired. Following excitation with the 405nM laser at a power of 50mW, the 488nM laser at a power of 200mW, the 561nM laser at a power of 100mW, and the 642nM laser at a power of 150mW, all cells were captured with 60x magnification (Numerical Aperture 0.9) using the enhanced depth field optic (EDF) at low speed (60mm per second) with an average rate of ~10 cell images per second. Emitted light was collected using the following filters: Channel 1 (Camera 1 420-480nm) and 9 (Camera 2 570-595nm) for brightfield (BF), channel 2 (Camera 1 480-560nm) for Alexa Fluor 488, channel 3 (Camera 1 560-595nm) for phycoerythrin (PE), channel 5 (Camera 1 660-740nm) for PerCP Cy5.5, channel 7 (Camera 2 420-505nm) for DAPI, and channel 11 (Camera 2 660-740nm) for AlexaFluor 647. All data were analyzed using IDEAS® software (version 6.2).

Image Compensation

Image compensation was conducted on samples that were exposed to CPT prior to being stained because they had the brightest H2aX foci. Cells stained with only DAPI, Tuj1-Alexa Fluor 488, H2AX-Alexa Fluor 647, Nestin-PerCP Cy5.5, and Active Caspase 3-PE were collected without BF illumination or Scatter and IDEAS® 6.2 was used to generate the compensation matrix.

Analysis of Cell Images and Calculation of Foci Number

Cells treated with CPT were analyzed first to determine a masking strategy and calculate foci number since they had the brightest and highest number of foci. The optimal fluorescence display for fluorescence imagery was determined by adjusting the image gallery properties in a linear fashion and applied to all images. To analyze the cells several steps and gating strategies were employed based on IDEAS® 6.2 software and incorporated wizards. The gates were created using the polygon gating tool and determined based on FMOs. The number of γH2AX foci were

quantified using a series of masks and quantification tools that were first tested on cells with brightest foci to ensure accurate foci counts and then applied to all samples and respective raw data files in batch processing.

Figure S1. Related to Figure 1 | hiPSC validation. hiPSC derived NPCs and Neurons express appropriate cell markers at each stage of reprogramming and differentiation. (A and B) Representative images of neurotypic BJ hiPSCs are positive for pluripotency markers Tra-181 and Oct 4. (C) Neural progenitor cells derived from hiPSCs show expression of NPC markers Nestin and Pax6. (D) Six-week neurons differentiated from NPCs are positive for neuronal markers Tuj1 and Map2. (E) Both neurotypic cell lines used in this study had normal karyotypes.

Figure S2. Related to Figure 2 | Nutlin treatment is lethal to wildtype NPCs. (A) Bright field images of NPCs show significant cell death in wildtype NPCs (pLKO.1) treated with Nutlin compared to other cultures. (B) Quantification of live cell count reveals complete cell death in wildtype NPCs treated with Nutlin, no difference between untreated controls and p53KD treated with Nutlin, and higher live cell counts in p53KD compared to controls (2-way ANOVA, Sidak's Multiple Comparisons Test, p<.0001).

Figure S3. Related to Figure 3 | Reproducibility of genome-wide DSB mapping/sequencing in 9429 NPCs and DNA DSBs and Gene Length. (A) Representative scatter plots of genome-wide DSB mapping/sequencing reads from two biological duplicates (N1 and N2) of the control (upper left), the APH-treated control (upper right), the $p53^{KD}$ (lower left) and the APH-treated-$p53^{KD}$ (lower right) samples treated for one day show strong correlations (Pearson's correlation r = 0.93-0.95, p ~ 0). Read-normalized DSB coverage for each preparation was calculated for 100 kb genome-wide, non-overlapping windows (n=30,895 bins); outliers (0.05% of total bins, 15 bins), bins with zero coverage, and centromeric/blacklisted regions were removed; Pearson's correlation was calculated. (B) NPCs treated for 1 day. DNA DSB read coverage is plotted against gene length in 5 bins based on all genes spanning the TSS +/- 2kb (n=25362 genes). No effect of $p53^{KD}$ or APH on DNA DSB location with respect to gene length. (C) NPCs treated for 4 days. DNA DSB read coverage is plotted against gene length in 5 bins based on all genes spanning the TSS +/- 2kb (n=25362 genes). APH Controls have significantly more DNA DSBs than DMSO controls at all gene lengths (* indicates Wilcoxon signed-rank test, two-sided, paired test, p<.00001). The box plot shows the 25th and 75th percentile; the middle line is the median; the whiskers span 5% to 95%, and outliers are not shown.

Figure S4. Related to Figure 3 | Genomic Distribution of DSBs. DNA DSBs were annotated uniquely to genomic regions: promoter, transcription start site (TSS), gene body (GB), transcription termination site (TTS), or intergenic region. (A) Pie-chart showing genome distribution of DSB density in control NPCs and p53KD after 24 hrs of Aphidicolin or DMSO treatment. (B) Pie-chart showing genome distribution of DSB density in control NPCs and p53KD after 4 days of Aphidicolin treatment.

Figure S5. Related to Figure 4 | p53 activation from transcriptional stress in individual neurotypic cell lines. (A) NPCs exposed to genomic stress (APH or CPT) had significantly more NPCs with DSBs than controls (One-way ANOVA, Dunnett's Test p<.0001). **(B)** BJ NPCs shown here have muted, but similar, levels of DNA DSBs relative 9429 (c.f., Fig 4A) (Chi-squared p<.001). **(C)** NPCs exposed to genomic stress are significantly more arrested in S-phase compared to controls (APH, Two-way ANOVA, Dunnett's Test p<.01, CPT, Two-way ANOVA, Dunnett's Test p<.05). **(D)** Representative western blot of NPCs exposed to stress for

24 hours. Original Blot presented in Supplementary Figure S7C. **(E)** Differential expression heatmap of the top five genes in each transcriptional cluster from scRNA-seq of NPCs stressed with CPT for 24 hours.

Figure S6. Related to Figure 5 | Gene Expression Levels of Nervous System Genes. Expression (in WT cells) of differentially broken TSSs between WT and $P53^{KD}$ were compared to total gene expression. Boxplot shows total gene expression in WT cells, red marks indicate differentially broken TSS genes annotated as neuronal, black marks indicate differentially broken TSS genes annotated as non-neuronal. (A) Full range of the gene expression data, including outliers. (B) Limited range of gene expression for the 61 differentially broken TSS genes. (C) Further limited range to better display 5th to 95th intervals of total expression.

Figure S7. Related to Figure 1, 2, and 4 | Uncropped Western Blots. Boxed regions indicate sections used to compile figure panels. (A) Western Blots correspond to experiments shown in Figure 1B. (B) Western Blots correspond to experiments shown in Figure 2A. Blots were first probed with rabbit anti-phosphorylated p53 and then with mouse anti-alpha tubulin after extensive washing. (C) Western Blots correspond to experiments shown in Supplementary Figure S5D.

Table S1. Sequencing and alignment statistics for paired-end Illumina NextSeq and HiSeq sequencing libraries prepared from NPC 9429.

Samples	Biological duplicates	# Sequenced read pairs[a]	% Alignment rate[b]	# Proper read pairs[c]	% Duplication[d]	# Mapped DSBs[e]	% Mapped DSBs[f]
1 day							
UT	N1	29,426,373	84.23	23,373,028	19.22	17,227,956	58.55
	N2	18,280,509	88.09	15,403,919	24.40	10,577,057	57.86
P53KD	N1	26,321,446	84.97	21,142,354	27.90	13,686,592	52.00
	N2	23,340,572	64.94	14,109,425	24.77	9,440,213	40.45
UT+APH	N1	31,503,611	89.44	26,997,237	22.56	19,214,999	60.99
	N2	27,034,866	82.76	21,133,279	21.59	15,153,850	56.05
P53KD+APH	N1	24,655,738	85.90	20,148,514	34.62	11,772,532	47.75
	N2	21,823,528	84.69	17,535,363	30.87	10,766,162	49.33
4 day							
UT	N1	23,669,751	96.53	22,121,971	23.51	15,603,469	65.92
P53KD	N1	16,838,730	95.73	15,612,040	27.64	10,304,419	61.19
UT+APH	N1	22,845,154	95.82	21,138,937	20.94	15,350,711	67.19
P53KD+APH	N1	23,149,237	96.39	21,560,888	23.97	15,012,066	64.85

[a] Number of raw paired read1-read2s following Illumina paired-end sequencing and quality filtering

[b] Percentage of reads that had at least one alignment to the hg38 genome assembly as processed and reported by bowtie2 alignment program

[c] Following the quality control removal of all unmapped, non-primary, supplementary and low-quality reads, the remaining number of paired read1-read2s are indicated.

[d] PCR duplicates are marked and removed meaningfully using read1 and read2 alignment, and “% Duplication” is based on original “# Sequenced read pairs”.

[e] For each non-duplicated pair, only read1 is kept, and the 5’ most nucleotide of read1 defines the DNA break position.

[f] “% Mapped DSBs” was calculated by dividing “# Mapped DSBs” by “# Sequenced read pairs”.

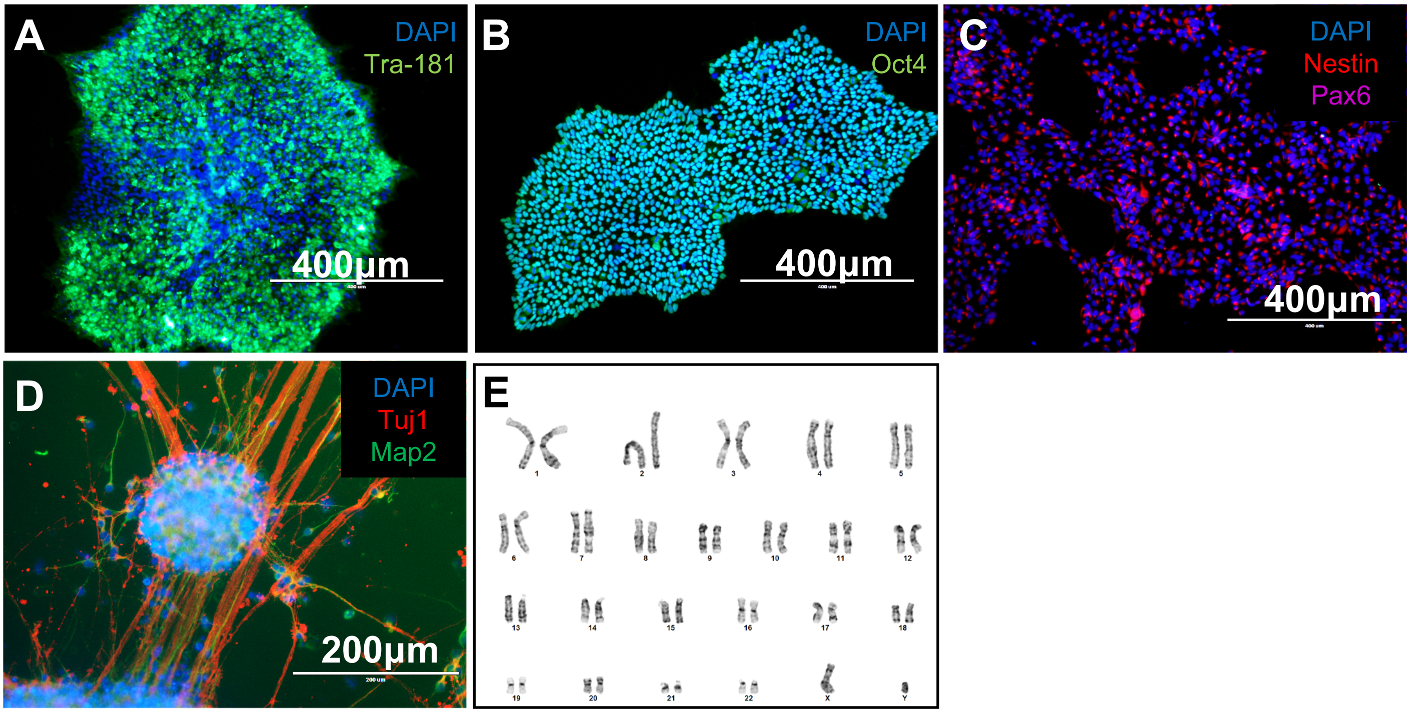

Figure S1. Related to Figure 1 | hiPSC validation

A

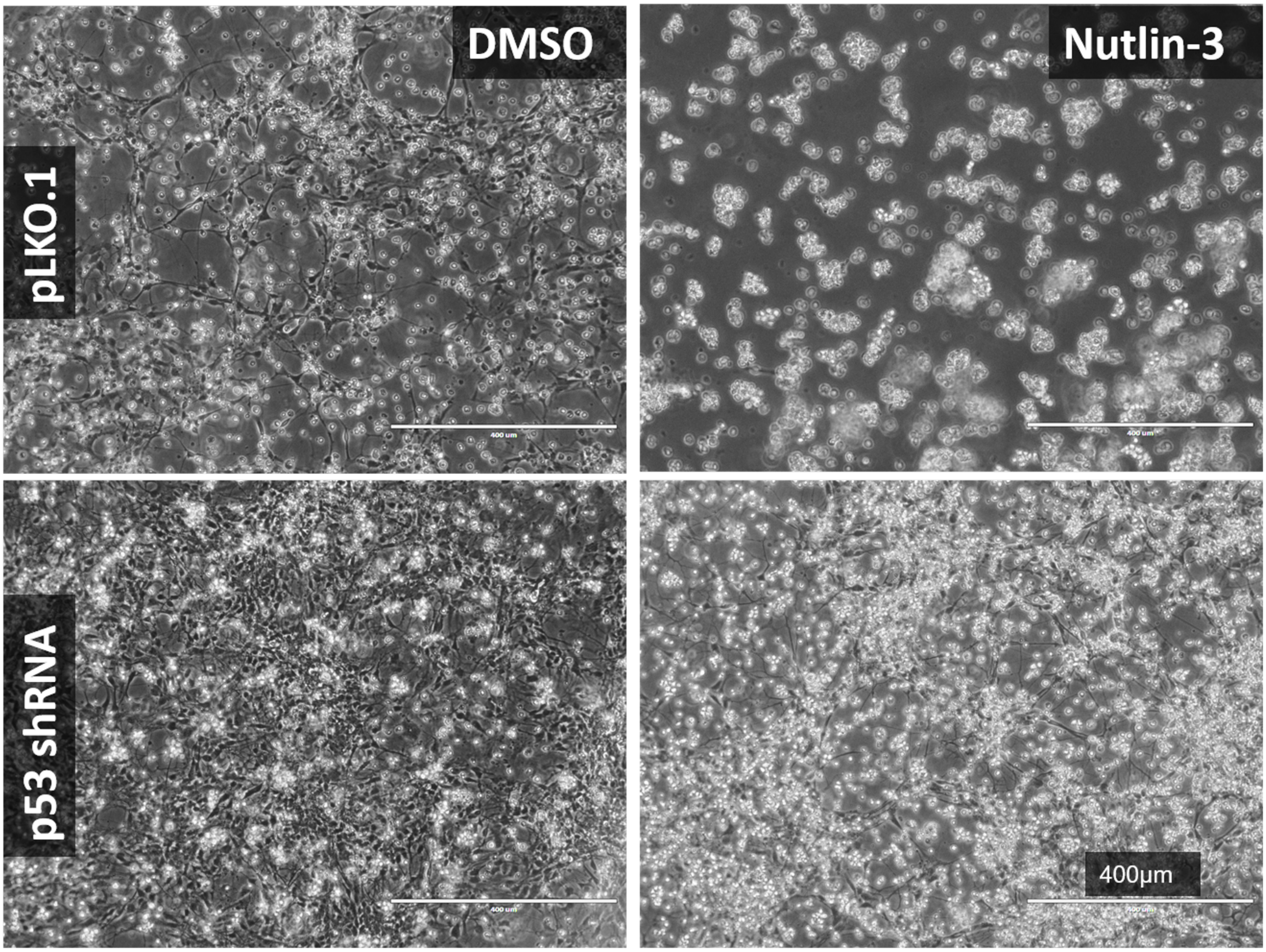

B

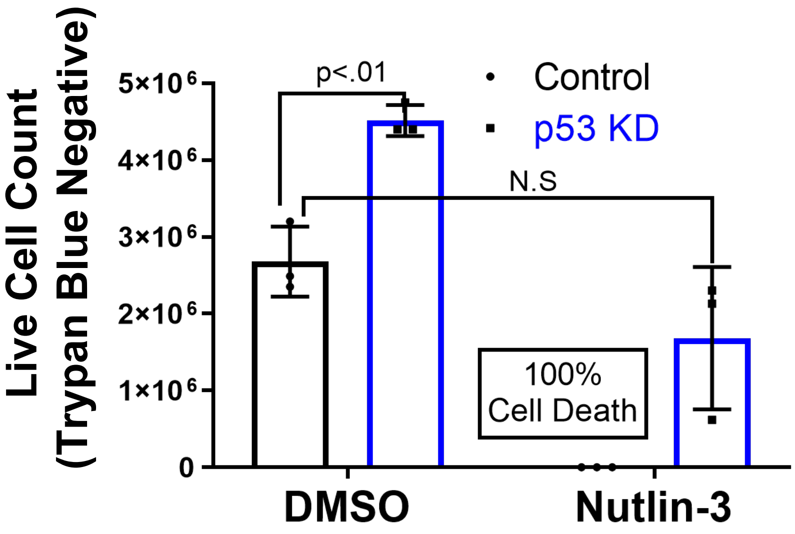

Figure S2. Related to Figure 2 | Nutlin treatment is lethal to wildtype NPCs

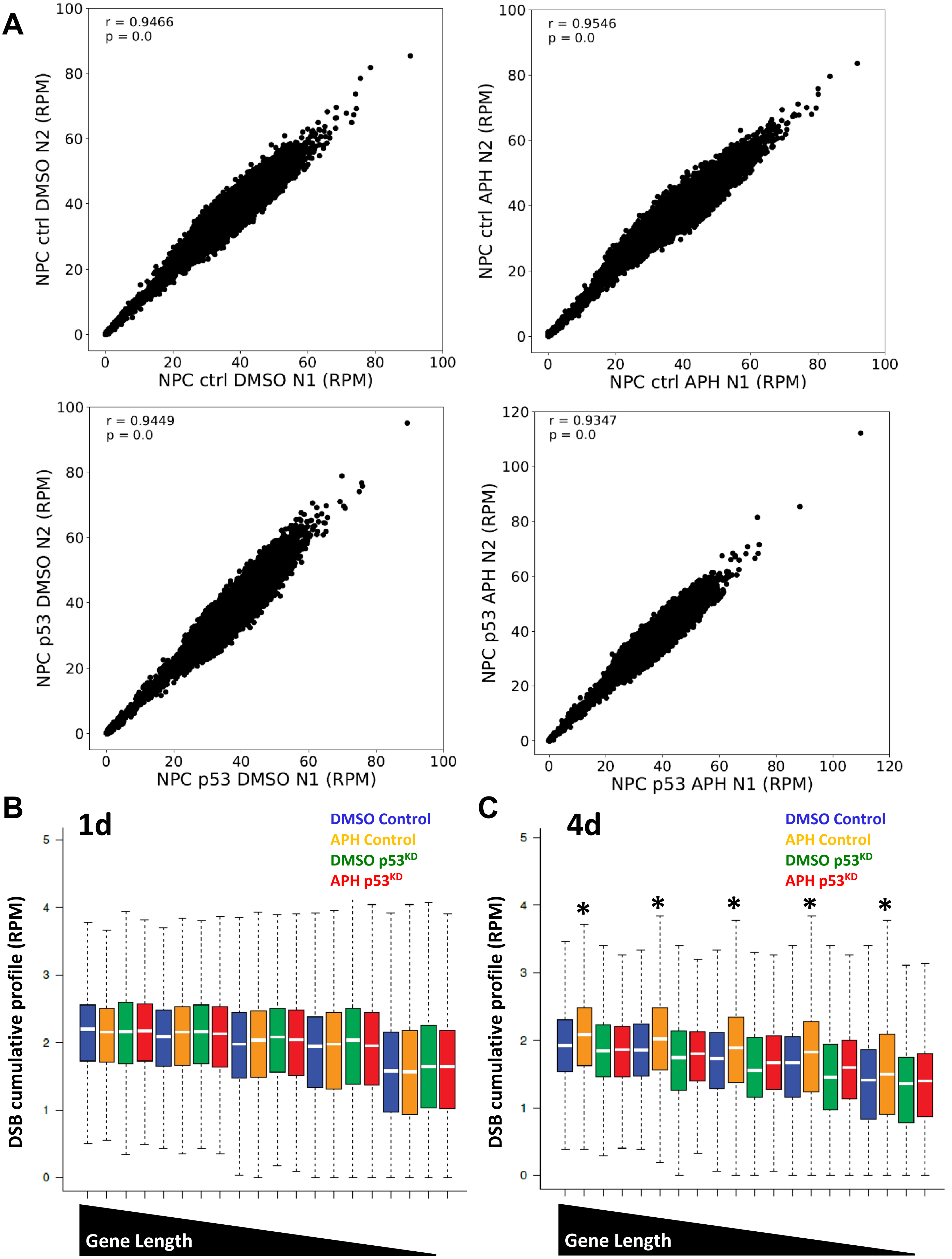

Figure S3. Related to Figure 3 | Reproducibility of genome-wide DSB mapping/sequencing in 9429 NPCs and DNA DSBs and Gene Length.

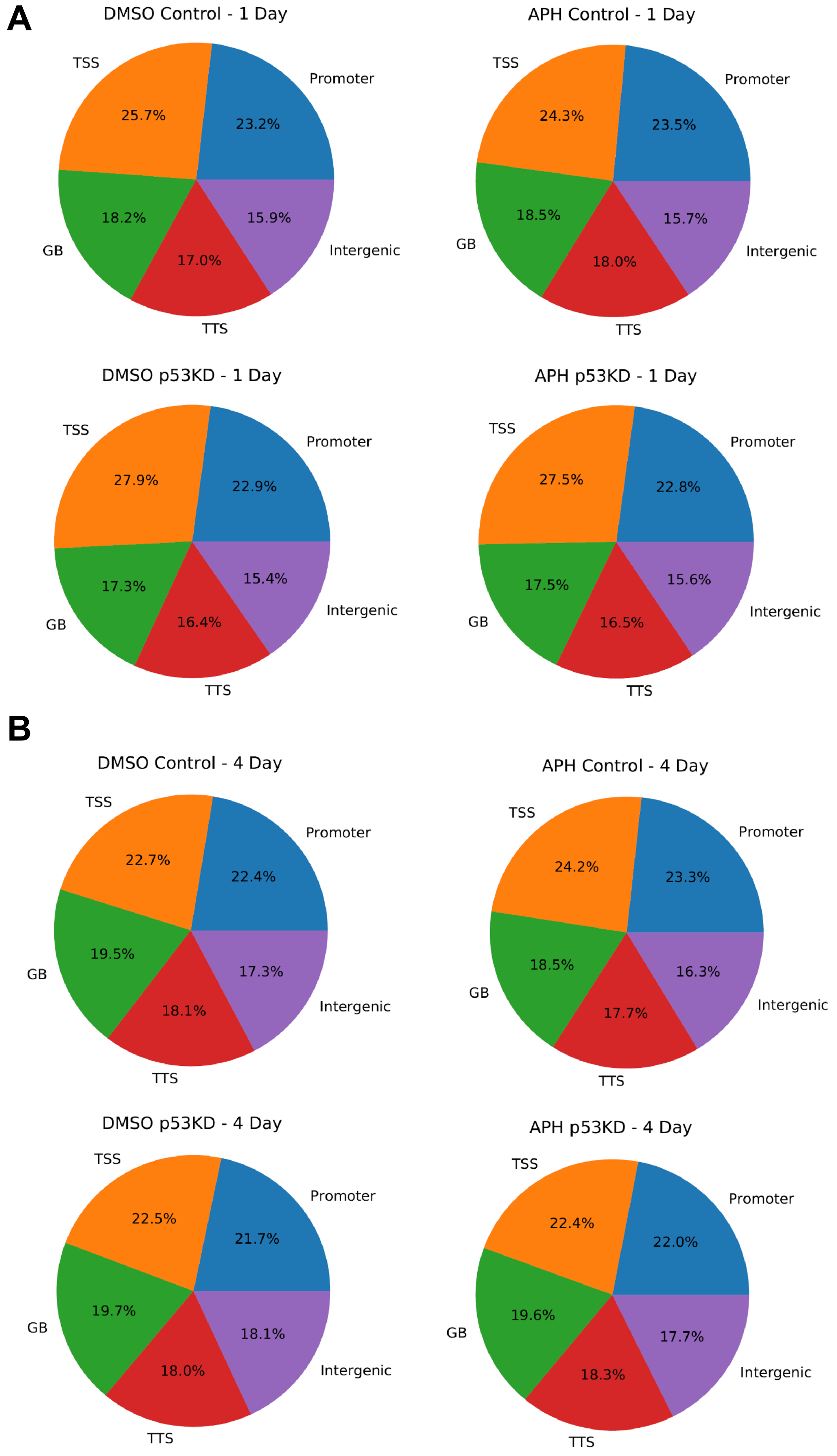

Figure S4. Related to Figure 3 | Genomic Distribution of DSBs.

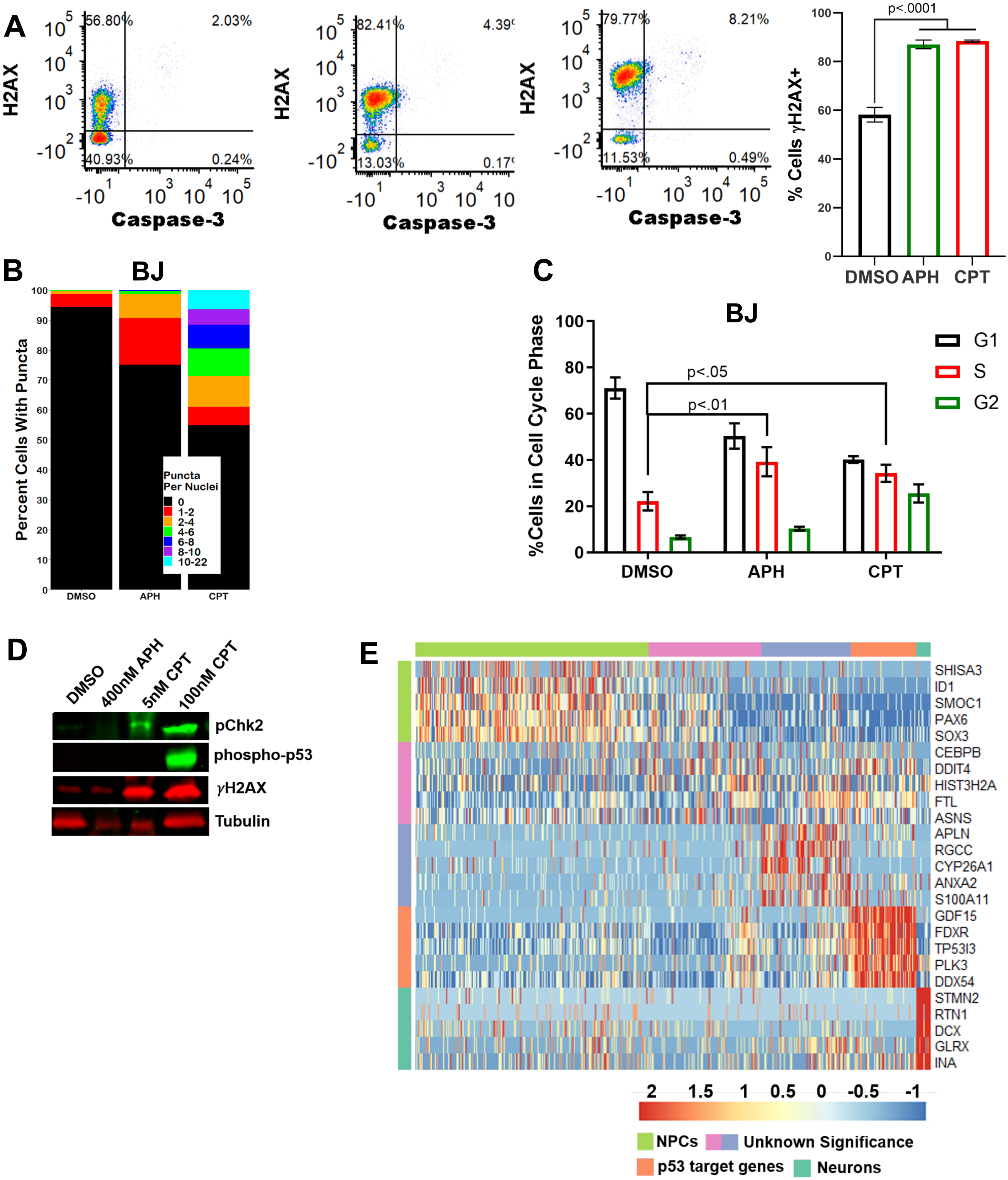

Figure S5. Related to Figure 4 | p53 activation from transcriptional stress in individual neurotypic cell lines.

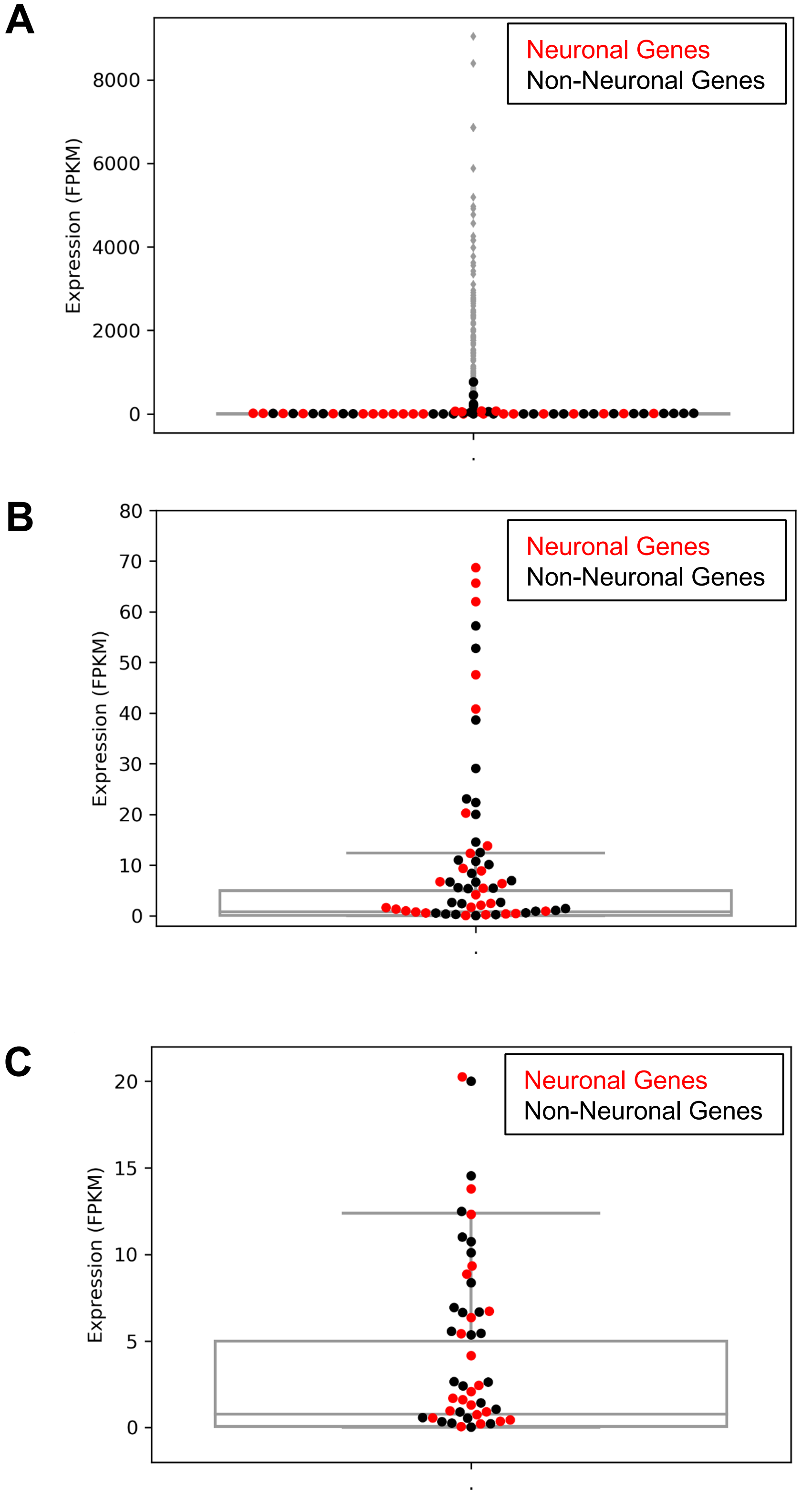

Figure S6. Related to Figure 5 | Gene Expression Levels of Nervous System Genes

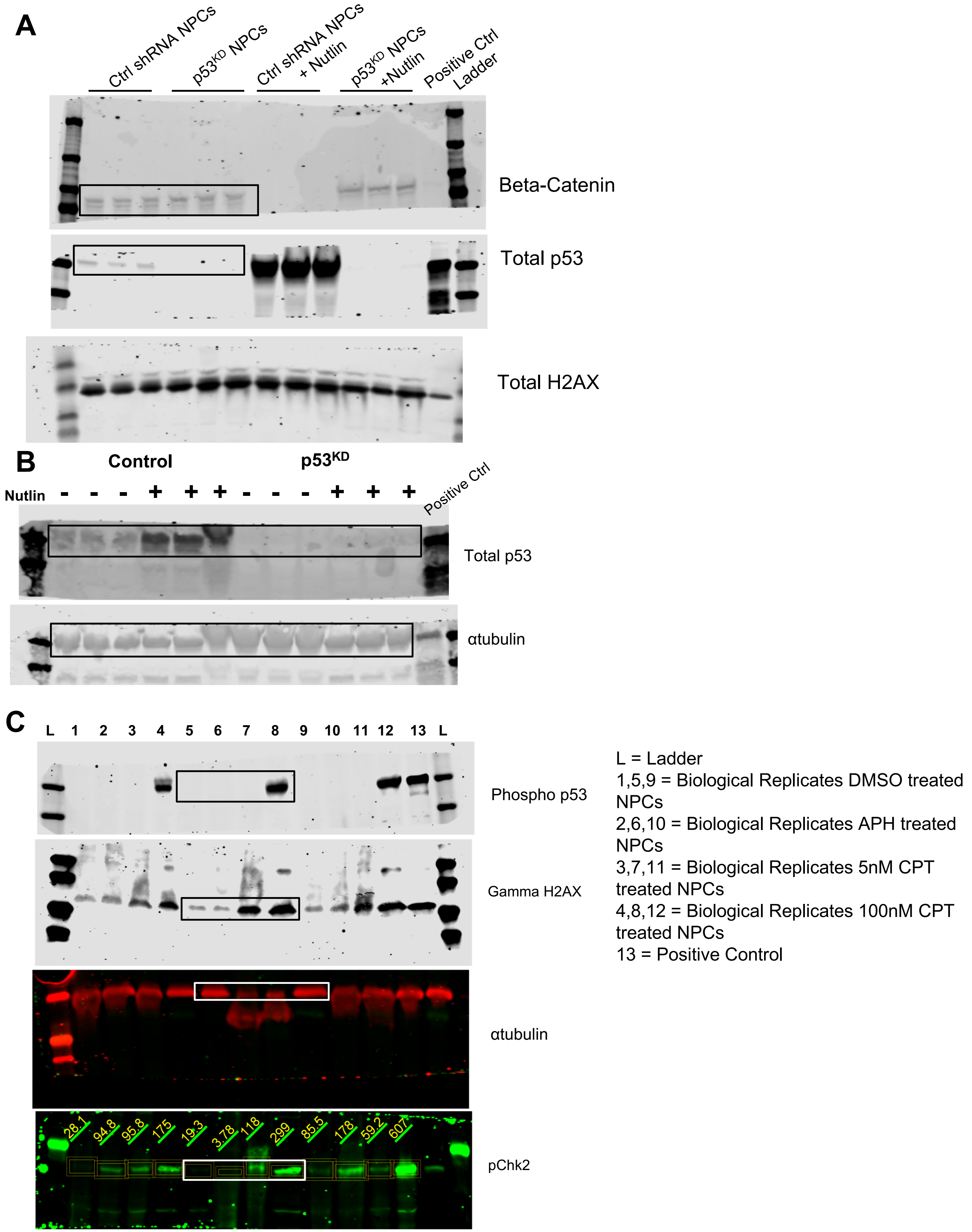

Figure S7. Related to Figure 1, 2, and 4 | Uncropped Western Blots
